# Convergent Spinal Circuits Facilitating Human Wrist Flexors

**DOI:** 10.1523/JNEUROSCI.1870-17.2018

**Published:** 2018-04-18

**Authors:** Stefane A. Aguiar, Stuart N. Baker

**Affiliations:** Institute of Neuroscience, Newcastle University, Newcastle upon Tyne NE2 4HH, United Kingdom

**Keywords:** Golgi tendon organ, H-reflex, Ib pathways, spinal circuitry, spinal cord

## Abstract

Noninvasive assessment of spinal circuitry in humans is limited, especially for Ib pathways in the upper limb. We developed a protocol in which we evoke the H-reflex in flexor carpi radialis (FCR) by median nerve stimulation and condition it with electrical stimulation above motor threshold over the extensor carpi radialis (ECR) muscle belly. Eighteen healthy adults (8 male, 10 female) took part in the study. There was a clear reflex facilitation at a 30 ms interstimulus interval (ISI) and suppression at a 70 ms ISI, which was highly consistent across subjects. We investigated the following two hypotheses of the possible source of the facilitation: (1) ECR Ib afferents from Golgi tendon organs, activated by the twitch following ECR stimulation; and (2) FCR afferents, from spindles and/or Golgi tendon organs, activated by the wrist extension movement that follows ECR stimulation. Several human and monkey experiments indicated a role for both of these sets of afferents. Our results provide evidence for a spinal circuit in which flexor motoneurons receive convergent excitatory input from flexor afferents as well as from extensor Ib afferents; this circuit can be straightforwardly assessed noninvasively in humans.

**SIGNIFICANCE STATEMENT** Here we described a novel spinal circuit, which is easy to assess noninvasively in humans. Understanding this circuit in more detail could be beneficial for the design of clinical tests in neurological conditions.

## Introduction

Assessing spinal circuitry noninvasively in humans is crucial for understanding both healthy function and pathology. For example, Delwaide (2001) suggests that the cause of rigidity in Parkinson's disease is a dysfunction in Ib pathways; a test to assess the function of these circuits would therefore have clinical interest. Unfortunately, methods for noninvasive assessments in humans are limited. The measurement of reciprocal inhibition, for instance, is often considered a very challenging protocol since it requires the stimulation of both median and radial nerves simultaneously and the recording of changes in the H-reflex according to the interval between stimuli (Day et al., 1984). Nevertheless, great progress in this field has been achieved with a number of existing and well established techniques to assess different circuits such as cutaneomuscular reflexes (Jenner and Stephens, 1982), recurrent inhibition due to Renshaw cells (Bussel and Pierrot-Deseilligny, 1977), and presynaptic inhibition (Berardelli et al., 1987), among others (for review, see Pierrot-Deseilligny and Burke, 2012).

Assessing Ib pathways in human upper limb has proved to be particularly challenging. Burne and Lippold (1996) used electrical stimulation percutaneously over tendons of different muscles to assess “autogenic” (nonreciprocal) inhibition of ongoing muscle activity, claimed to be caused by a polysynaptic pathway originated from Ib afferents from Golgi tendon organs. However, Priori et al. (1998) criticized not only the long latency of this inhibition, which was not compatible with an Ib pathway, but also the location of stimulation over tendons since Golgi tendon organs are actually located in the musculo-tendinous junction (Jami, 1992). Ultimately, this inhibition is more likely generated by slowly conducting group III tendon afferents producing presynaptic inhibition of Ia fibers (Priori et al., 1998). Another potential test to assess Ib pathways was described by Cavallari et al. (1985), who demonstrated a facilitation of the flexor carpi radialis (FCR) H-reflex by a prior radial nerve stimulus at an interval of 2 ms. They suggested that this was mediated by Ib pathways and enhanced by cutaneous stimulation. However, as noted by the authors themselves, in most cases this Ib facilitation was less clear, manifesting only as an inflection superimposed on the decay of the shorter-latency inhibition. The lack of consistency makes this protocol not ideal for assessing the function of Ib pathways in humans.

In this study, we used median nerve stimulation to evoke an H-reflex in the FCR muscle, and conditioned this by previous electrical stimulation above motor threshold to the extensor carpi radialis (ECR) muscle belly. With a 30 ms interstimulus interval (ISI), we found a clear facilitation. Two hypotheses were investigated as the source of this effect: (1) ECR Ib afferents from Golgi tendon organs, activated since the high-intensity ECR stimulation causes a twitch, pulling on the ECR tendon; and (2) FCR afferents, from muscle spindles and/or Golgi tendon organs, activated by the wrist extension movement that follows from the ECR contraction. Results from a series of experiments in both human subjects and monkeys supported the participation of both ECR Ib afferents and FCR afferents in the effect. Our work reveals novel convergent spinal circuits not yet described in the literature, with flexor motoneurons receiving convergent input from FCR afferents and ECR Ib afferents, which can be easily assessed noninvasively in humans.

## Materials and Methods

### 

#### Experiments in healthy human subjects

**Figure 1.**
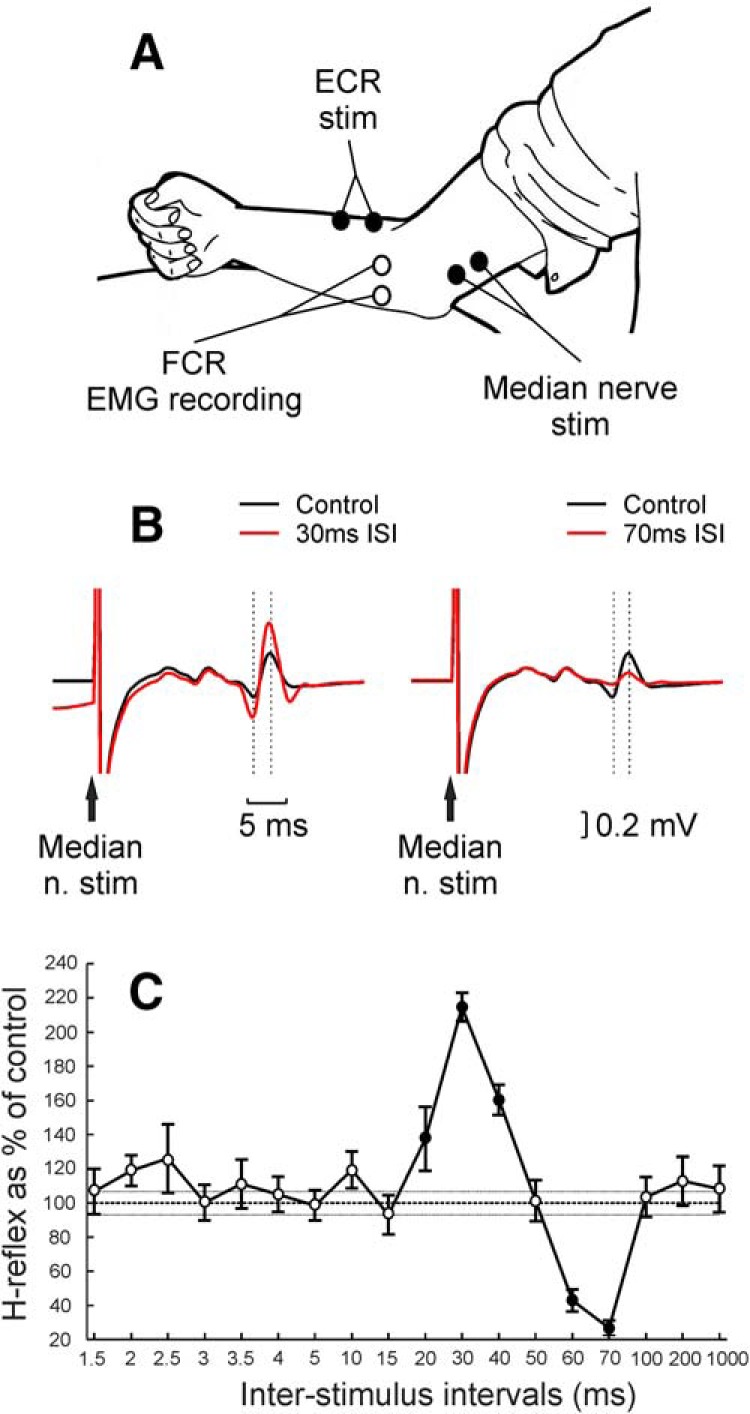
Experimental setup and example of main result in a healthy human subject. ***A***, Schematic representation of experimental setup. EMG recording electrodes were positioned over FCR muscle belly, and stimulating electrodes were positioned over the median nerve (to evoke FCR H-reflex) and over the ECR muscle belly. ***B***, Example from one subject of the comparison between control (black) and conditioned (red) H-reflex at both 30 and 70 ms ISIs between median nerve stimulation (n. stim) and conditioning ECR electrical stimulation at 3× MT; traces are the average of 20 repetitions for control and 10 repetitions for conditioned H-reflex. Dashed lines indicate the times used to measure H-reflex amplitude. ***C***, Example from the same subject of conditioning curve showing the H-reflex amplitude as a percentage of its control amplitude for 18 ISIs between median nerve and conditioning ECR electrical stimulation at 3× MT. Filled circles represent responses significantly different from control. Error bars represent the SE. Thin dashed horizontal lines indicate the SE of control H-reflex amplitude.

Eighteen healthy human volunteers, 18–56 years of age, participated in this study (8 male and 10 female). All subjects signed a written consent form before participation, and all procedures were approved by the Ethical Committee of the Medical Faculty, Newcastle University. Subjects were seated comfortably on a chair with their forearm resting on a table. To measure and evoke the H-reflex, electromyography (EMG) recording electrodes (Kendall H59P, Medcat) were placed on skin overlying the FCR muscle belly, with a reference over the brachioradialis, and connected to a Digitimer NL 824 amplifier (gain, 2000; bandpass, 30 Hz to 2 kHz). A stimulating electrode (bipolar felt pad, P20–4zl, Medcat) was placed over the median nerve just above the elbow (cathode proximal) and connected to an isolated constant-current stimulator (model DS7A, Digitimer; model DS5, Digitimer, for vibration and threshold protocols). Electrode placement was accomplished according to the study by Jabre (1981). The response was identified as an H-reflex if it had a latency between 12 and 25 ms, an amplitude that increased during voluntary contraction, and the amplitude fluctuated with changes in stimulus intensity together with the M-wave amplitude (all as described in Jabre, 1981). The recordings were made with the amplitude of an H-reflex of ∼50% of the maximum observed for each subject. We refer to these responses for simplicity as the “FCR H-reflex,” but note here that with surface recording electrodes there may be some contribution from other nearby muscles. Background EMG activity was small or negligible since the muscle was at rest. The stimulating electrodes on the median nerve were kept fixed by a strap wrapped around the subject's arm (monophasic pulse, intensities up to 10 mA, 500 μs pulse width). Stimulating electrodes were also placed on the ECR muscle belly for the conditioning stimuli, with the proximal cathode stimulating at an intensity 3×that of the motor threshold (MT; monophasic pulse, intensities up to 24 mA, 1 ms pulse width). This intensity was chosen as it was strong enough to generate clear effects, but was not uncomfortable for the subjects. An assessment of the effect of changing the intensity is reported in Results. MT was defined as the intensity that produced the first visible twitch of the ECR muscle. A schematic representation of electrode positioning is shown in Figure 1*A*.

During all protocols, subjects were instructed to remain relaxed, with no contraction of forearm muscles, and to maintain their arm in a constant position. The lack of activity in the FCR recording was constantly monitored by the experimenter. Subjects were randomly selected to take part in the different experimental protocols described subsequently.

##### Main protocol.

The H-reflex was conditioned by previous stimulation of ECR at the following 18 ISIs: 1.5, 2, 2.5, 3, 3.5, 4, 5, 10, 15, 20, 30, 40, 50, 60, 70, 100, 200, and 1000 ms. Each combination of test and conditioning stimuli was separated by an interval of 4 s, and the amplitude of the H-reflex was expressed as a percentage of the control H-reflex (with no conditioning ECR stimulation). Ten repetitions of each ISI and 20 repetitions for the control H-reflex were completed, delivered in pseudo-random order. Reflex amplitude was measured and was expressed as a percentage of the control values for each subject. A two-way ANOVA with the factors subjects and ISI was performed to investigate whether conditioning ECR stimulation had any effect on H-reflex measurements. If the ANOVA showed a significant effect of interval, we tested which ISIs showed significant differences from 100% across subjects using *t* tests, with a significance level of *p* < 0.05. A similar approach was used to assess reflex changes in all of the experiments listed below. For the ISIs of 30 and 70 ms, we also conducted a separate analysis for each subject using *t* tests on the single-sweep reflex amplitudes to determine how many subjects showed significant facilitation (30 ms ISI) and/or inhibition (70 ms ISI); the significance level was set as *p* < 0.05. Data from 17 subjects were gathered for this protocol.

##### Threshold intensity.

In six subjects, we tested 11 intensities of ECR stimulation (1, 1.2, 1.4, 1.6, 1.8, 2, 2.2, 2.4, 2.6, 2.8, and 3× MT) only for the ISI of 30 ms.

##### Threshold changes after vibration.

In five subjects, we tested the H-reflex conditioned with an ISI of 30 ms at five intensities of ECR stimulation (1.5, 1.8, 2.1, 2.4, and 2.7× MT) before and after vibration for 25 min over the ECR tendon (166 Hz). Subjects were instructed to remain relaxed during the vibration, and no overt contractions of ECR were observed.

##### ECR twitch time.

In nine subjects, stimuli were delivered to the ECR muscle at 3× the MT while subjects had their forearm held in a horizontal position (wrist 90° from the horizontal plane) and grasped a metal bar attached to a strain gauge, which detected force in the direction of flexion-extension. For each subject,we recorded 64–122 repetitions of the stimulus, and averaged the poststimulus force profile. The time of the first peak in force production after the ECR stimulation was measured and referred to as the twitch time.

##### ECR tendon taps.

In nine subjects, we replaced the conditioning stimulus with a mechanical tap applied directly to the ECR tendon instead of electrical stimulation, with the same 18 ISIs used previously. The tap apparatus (LDS V201 shaker with PA25E power amplifier, Brüel & Kjaer) was positioned above the ECR tendon ∼3–4 cm more proximal than the wrist, with the tapper applying light pressure on the skin. The tap intensity was at tap threshold (TT), defined as the lowest tap amplitude that produced a visible response in the ECR muscle EMG.

##### FCR tendon taps.

This protocol was identical to that described above, except that the mechanical tap was applied to the tendon of the FCR muscle (∼3–4 cm more proximal than the wrist). Data from five subjects were gathered for this protocol.

##### Cutaneous stimulation.

In five subjects, we tested the impact of cutaneous stimuli on the H-reflex facilitation. Cutaneous stimulation (200 μs pulse width; intensity, 2× the perceptual threshold; DS7A stimulator, Digitimer) was given through surface electrodes on the dorsal side of digits 2 and 3 (Cavallari et al., 1985). The H-reflex elicited by the median nerve stimulus was tested with the following three conditioning conditions: (1) H-reflex plus ECR stimulation (30 ms ISI); (2) H-reflex plus cutaneous stimulation (25 ms ISI); and (3) H-reflex plus ECR stimulation plus cutaneous stimulation (both timed relative to the median nerve, as described above). Forty repetitions of each condition and of the control H-reflex were typically measured.

##### Wrist flexed.

In eight subjects, the main protocol described above was repeated while subjects had their wrist held in a flexed position by a Velcro strap. The degree of wrist flexion adopted was the maximum possible in that subject. In this position, the wrist flexors should have been slack, reducing or eliminating the activation of flexor muscle afferents following the ECR twitch. Wrist flexion was achieved passively by the strap, with no active flexion by the subject, as judged by the absence of background EMG in the FCR recording.

##### Passive movement.

In six subjects, the conditioning stimulus was replaced by a passive extension movement around the wrist joint, generated by a torque motor. The extension movement was notably larger than that typically generated by ECR electrical stimulation in the main protocol. The maximum amplitude of the passive movement for the subjects tested was 14.2 ± 1.5°, and the time to reach maximum movement amplitude was 110.7 ± 6.5 ms. After the point of maximum extension, the wrist joint returned to its neutral position; the total movement duration was 190.5 ± 13.1 ms (mean ± SD). Note that according to the duration of the main facilitatory effects observed in this study (see Results section), only the first few degrees of movement are likely to be relevant.

Most subjects volunteered for several protocols on the same day, although some subjects returned on multiple days; the numbers of subjects tested in each protocol are listed above. Electrodes were placed consistently within the same subject across days, but any minor differences would not affect the results, as pairwise comparisons were not made between different protocols.

#### Monkey experiments

All animal procedures were performed under UK Home Office regulations in accordance with the Animals Scientific Procedures Act (1986) and were approved by the Animal Welfare and Research Ethics Board of Newcastle University. Recordings were made from four terminally anesthetized female rhesus macaque monkeys (coded PLK, PAT, PDR, and TNS in this study: age, 9.5, 9.6, 9.8, and 7.1 years; weight, 10.4, 9.5, 9.3, and 6.4 kg, respectively).

##### Initial surgical preparation.

Animals were initially sedated by intramuscular injection of ketamine (10 mg · kg^−1^); then deep anesthesia was induced with propofol (5–10 mg · kg^−1^, i.v.). Anesthesia was then switched to sevoflurane inhalation (1.5–3%) and an intravenous infusion of alfentanil (11–18 μg · kg^−1^ · h^−1^) with artificial ventilation. Central arterial and venous lines were inserted via the external carotid artery and external jugular vein, and a tracheotomy was made to secure the airway. The bladder was catheterized. Anesthetic monitoring included central arterial and venous pressure, heart rate, blood oxygen saturation, end-tidal carbon dioxide, and core and peripheral temperatures. The animal was kept warm with a thermostatically controlled heating blanket, and a system that surrounded the animal with warmed air (Bair Hugger, 3M). Fluids were provided via a continual intravenous infusion (total rate with drug infusions, 5–10 ml · kg^−1^ · h^−1^).

##### Stimulation.

During the experiments the anesthetic regimen was switched to intravenous infusions of midazolam (270–460 μg · kg^−1^ · h^−1^), alfentanil (13–26 μg · kg^−1^ · h^−1^), and ketamine (5.8–9.0 mg · kg^−1^ · h^−1^); this regimen was chosen to increase the excitability of the nervous system (Witham et al., 2016). The H-reflex was often difficult to obtain in these animals due to the effects of anesthesia, and also its short latency sometimes made the H-reflex and M wave difficult to distinguish. We therefore used responses to pyramidal tract (PT) stimulation as a way of assessing the excitability of motoneurons in FCR. Fine tungsten stimulating electrodes (LF501G, Microprobes) were implanted into the PT on the side contralateral to the arm being tested. PT electrodes were positioned with reference to antidromic volleys recorded epidurally from motor cortex after stimulation through them (Riddle and Baker, 2010). Trains of two to four stimuli through the PT electrode, given 3 ms apart, were necessary to evoke an FCR response (2, 4, 3, and 4 trains of stimuli for monkeys PLK, PAT, PDR, and TNS, respectively; biphasic pulse; intensities up to 4 mA; 200 μs pulse width; Isolated Pulse Stimulator Model 2100, A-M Systems). For PT responses, unlike the H-reflex, homosynaptic depression is not a concern. The interval between each pair of test and conditioning stimuli was therefore reduced to 500 ms, instead of the 4 s used for human protocols, to accelerate data acquisition in these time-limited terminal experiments. The amplitude of the PT response was measured as the area under the curve of the rectified EMG signal from the FCR. For the conditioning stimulus to ECR, we used fine wire electrodes made of seven-stranded stainless steel wires insulated with Teflon and bared for a few millimeters at the tip (part number FE6320, Advent Research Materials); these were introduced by a needle, which was then withdrawn. In monkeys PLK, PAT, and PDR, the muscle was exposed and dissected first. Biphasic stimuli were given through these wires (intensity up to 6 mA; 1 ms pulse width for each phase; a second stimulator, as described above, A-M Systems). The forearm was fixed through screws attached to the bones (ulna and radius), near the elbow and wrist, for monkeys PLK, PAT, and PDR. Wire electrodes were also inserted into the FCR for EMG recording (NL824 amplifier, Digitimer; gain, 500–1000; bandpass, 30 Hz to 2 kHz). A schematic representation of electrode positioning is shown in Figure 5*A*.

##### Main protocol.

A protocol similar to the main protocol described above for human subjects was delivered to the four monkeys, with PT stimulation used as the test stimulus and ECR stimulation at 3× MT as the conditioning stimulus. The amplitude of the PT response was expressed as a percentage of the control PT response (with no conditioning ECR stimulation) for all monkey experiments. The same 18 ISIs as in the human studies were used except for the two largest, which were reduced to 150 and 200 ms. In the monkey recordings, ISIs refer to the delay from the conditioned stimulus to the last (effective) stimulus in the train delivered to the PT. A minimum of 20 repetitions of each ISI and 40 repetitions of the control PT response were completed.

##### ECR tendon cut.

We tested the main protocol in monkeys PLK, PAT, and PDR after cutting the ECR tendon near the wrist. The cut end of the tendon was left free.

##### FCR tendon cut and reattached.

We tested the main protocol in monkey TNS after cutting the FCR tendon near the wrist. Subsequently, we reattached it using a suture around the tendon sewn to the skin of the hand, maintaining tendon tension similar to that with the tendon intact.

##### ECR tendon pulls at large amplitude.

In monkey PDR, after the ECR tendon was cut, we used a surgical suture around the lose tendon to attach it to a mechanical puller (305C-I, Aurora Scientific), maintaining a similar resting muscle tension as before the tendon was cut. Brief mechanical pulls (2 mm amplitude, 1 ms duration) were used as conditioning stimuli preceding PT stimulation. The same 18 ISIs as in the main protocol were used, except that in this case the intervals were defined relative to the first stimulus in the train to the PT.

##### ECR tendon pulls at variable amplitude.

This was similar to the preceding protocol, except that tendon pulls of different intensities were used (20, 40, 60, 80, 100, 120, 140, 160, 180, 200, 250, 300, 350, 400, 450, 500, 750, 1000, 1500, and 2000 μm; all 1 ms duration). A single ISI of 2.5 ms, which had produced a clear facilitation with a 2 mm pull amplitude, was used.

All data analysis was performed using custom scripts written in the MATLAB environment (version R2015a).

## Results

### Experiments in healthy human subjects

Figure 1*B* shows an example from one subject of the control and conditioned H-reflex with the ISIs of 30 and 70 ms, which produced evident facilitation and suppression, respectively. The facilitation at the 30 ms ISI was significant in 13 of 17 participants, and the suppression at the 70 ms ISI was significant for all 17 volunteers (*p* < 0.05). Figure 1*C* depicts the H-reflex amplitude curve for the 18 conditioning ISIs from the same subject. It shows the typical pattern of facilitation followed by suppression, which was a consistent finding. Of the four subjects without significant facilitation at the 30 ms ISI, three showed reflex amplitudes close to control; one had a reflex that was 145% of control, but with a high variability (SEM, 25.7%) that prevented it from reaching significance. Facilitation was not seen at any other intervals for these four subjects.

**Figure 2.**
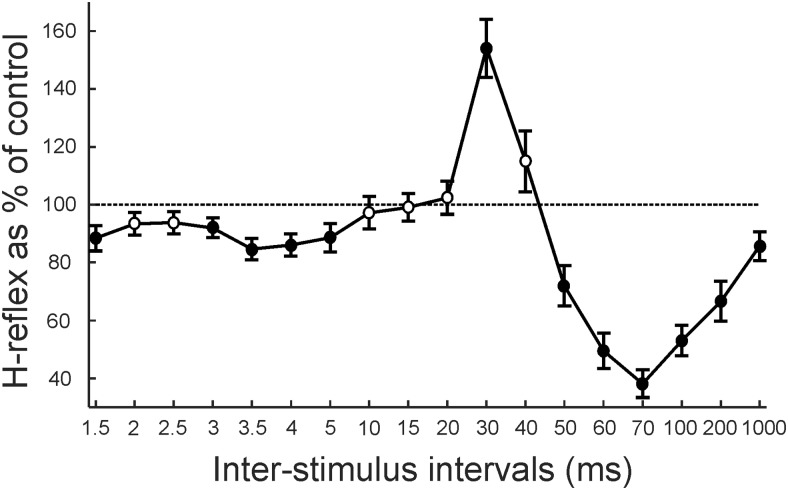
Population-averaged data in healthy humans. Average from 17 subjects of H-reflex amplitude, shown as a percentage of its control amplitude, for 18 ISIs between median nerve and conditioning ECR electrical stimulation at 3× MT. Filled circles represent responses significantly different from control. Error bars represent the SE. For one subject, data from ISIs up to 5 ms, and for another subject up to 3 ms, were omitted from the average due to contamination by the stimulus artifact.

Figure 2 shows the average results across subjects from the main protocol. There was a significant effect of ISI (ANOVA, *F*_(17,261)_ = 22.08, *p* < 0.001). Significant inhibition was found at ISIs of 1.5 and 3–5 ms (*t* test, all *p* < 0.05), significant facilitation at the ISI of 30 ms (*p* = 0.0001), and significant inhibition at ISIs of 50–1000 ms (*t* test, all *p* < 0.020). Subsequently, we conducted a number of different experimental protocols to investigate the origin of the facilitation observed at the 30 ms ISI.

**Figure 3.**
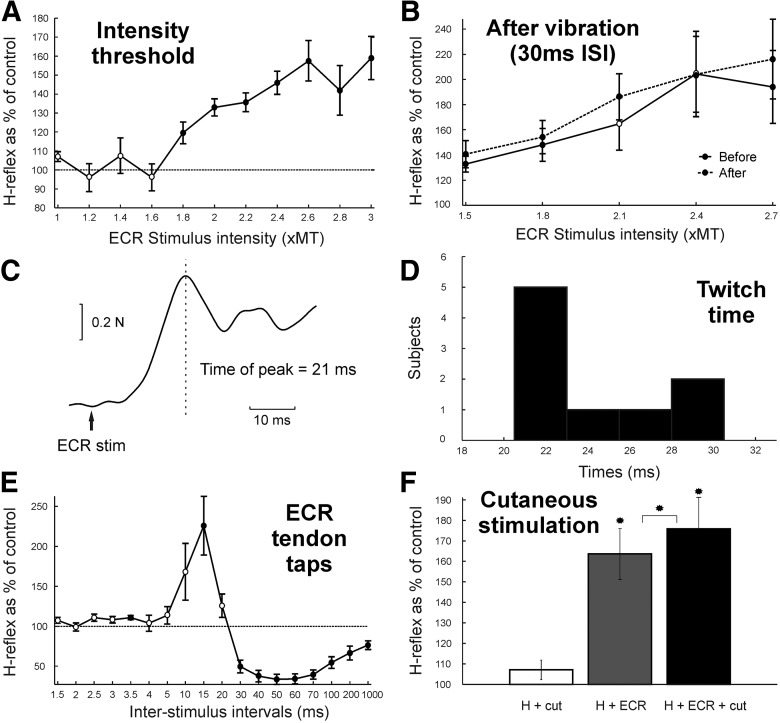
Studies in healthy humans probing which afferents mediate the facilitation. ***A***, Average from six subjects of H-reflex amplitude with 11 intensities of conditioning ECR electrical stimulation at the ISI of 30 ms between median nerve and conditioning ECR electrical stimulation. ***B***, Average from five subjects of H-reflex amplitude with five intensities of conditioning ECR electrical stimulation at 30 ms ISI between median nerve and conditioning ECR electrical stimulation. Continuous line shows measurements before vibration of ECR tendon for 25 min, and dashed line after vibration of ECR tendon for 25 min. ***C***, Example from one subject of force production from ECR muscle following stimulation at 3× MT. The time of the first observed peak in force production is indicated by the dashed line and taken as the measure of twitch time. ***D***, Distribution of twitch time measured in nine subjects. ***E***, Average from nine subjects of H-reflex amplitude following mechanical tap to ECR tendon as conditioning stimulation at 1× TT for 18 ISIs. ***F***, Average from five subjects of H-reflex amplitude (as a percentage of control) conditioned by cutaneous stimulation (2× perceptual threshold at the 25 ms ISI) alone (white bar), by ECR stimulation (3× MT at 30 ms ISI) alone (gray bar), and by ECR stimulation combined with cutaneous stimulation (black bar). *Significant differences. Display conventions in ***A***, ***B***, and ***E***, as in Figure 2. H + cut, H-reflex and cutaneous stimulation; H + ECR, H-reflex and ECR stimulation; H + ECR+ cut, H-reflex and ECR stimulation and cutaneous stimulation.

One hypothesis for the generation of this facilitation is that the stimulus-evoked twitch in ECR pulls on the extensor tendon, activating the Golgi tendon organs that mediate the effect. We first explored the ECR stimulus intensity required to generate the facilitation; results averaged across subjects are shown in Figure 3*A*. There was a significant effect of ECR intensity (ANOVA, *F*_(10,50)_ = 7.48, *p* < 0.001). A significant facilitation at the 30 ms ISI was present with ECR conditioning stimuli of ≥1.8× MT (*t* test, *p* = 0.0263). By definition, 1× MT produces a just-noticeable muscle twitch, although such stimuli are clearly above the perceptual threshold. The lack of H-reflex facilitation at weaker intensities is thus consistent with the generation by ECR Ib afferents.

Another possibility for the origin of the facilitation is Ia afferents from ECR, which could be directly activated by the stimulus to the motor point (Muir and Lemon, 1983). To investigate this, we repeated the measurement of the threshold intensity for ECR stimulation to generate the facilitation after a sustained period of vibrating the ECR tendon (25 min, 166 Hz). Such vibration has been proven to increase the threshold for electrical activation of Ia afferents, bringing it to a level higher than the threshold for Ib activation, which does not change (Coppin et al., 1970; Fetz et al., 1979; Katz et al., 1991). If Ia afferents were in fact the source of the facilitation, then at any stimulus intensity a greater proportion of fibers would be below threshold after sustained vibration. This would lead to a rightward shift of the curve relating facilitation to stimulus intensity. Figure 3*B* depicts the results of this experiment. The slight differences in the relationship with intensity between these data and Figure 3*A* are likely to reflect interindividual differences in activation at different percentages of MT; only two subjects participated in both experiments. Sustained vibration had no significant effect on the facilitation (three-way ANOVA with factors subjects, intensity, and before/after vibration; vibration, *F*_(1,490)_ = 3.37, *p* > 0.05).

It remains possible that vibration did exert an effect, but that this was too small to be detected within the statistical power of our measurements. To check for this, we measured the SD of the H-reflex for each subject at each conditioned intensity and also fitted a sigmoid curve to the results before vibration shown in Figure 3*B*. We then generated simulated data, where H-reflex amplitudes before vibration were modeled as Gaussian random numbers, with a mean that followed the sigmoid curve and an SD determined from each subject's original data. Data after vibration were similarly generated, except that the sigmoid curve was shifted to the right by a value Δ*I*. As in the actual data, points were generated for *n* = 5 subjects. We tested for significant differences between these simulated curves, using a three-way ANOVA exactly as applied for the experimental data. The process was repeated 1000 times at each Δ*I*, for Δ*I* from 0 to 0.5×MT in steps of 0.01 × MT. A significant difference between the curves was detected >90% of the time at *p* < 0.05 for shifts of Δ*I* > 0.33 × MT. Katz et al. (1991) showed a recruitment curve rightward shift of ∼0.3× MT following sustained vibration, which we estimate would have been detected from our data 83% of the time. We therefore conclude that electrical activation of ECR Ia afferents is unlikely to play a major role in the facilitation of the FCR H-reflex that we describe.

If the stimulus delivered to ECR generates the H-reflex facilitation via the mechanical consequences of the shock, the muscle would have to generate enough force to activate Ib afferents after <30 ms, to allow time for the afferent activity to reach the spinal cord and cause a central effect. Figure 3*C* illustrates the profile of force produced after the ECR stimulus for one subject; the first peak of the twitch tension occurred in this individual 21 ms after the stimulus. Figure 3*D* shows a histogram of ECR twitch time across nine subjects. The mean ± SD twitch time was 24.4 ± 3.5 ms. This timing is therefore appropriate to generate an H-reflex facilitation at the 30 ms interval.

To explore further the possible involvement of the mechanical activation of ECR Ib afferents, we tested the effect of replacing the ECR electrical stimulation by a mechanical tap to the ECR tendon at the threshold required to generate a tap reflex in the EMG of the resting ECR muscle (1× TT). As the muscle was at rest, this intensity would be substantially above the threshold for the activation of Ia fibers, and both Ia and Ib afferents should be activated (Lundberg and Winsbury, 1960; Katz et al., 1991). Figure 3*E* shows results averaged across all subjects. There was a significant effect of ISI (ANOVA, *F*_(17,136)_ = 12.56, *p* < 0.001). Significant facilitation was observed at the 3.5 and 15 ms ISIs (*t* test, *p* = 0.0057 and *p* = 0.0121, respectively) and significant inhibition at the 30–1000 ms ISIs (*t* test, all *p* < 0.007). Thus, although a tap to the ECR tendon could generate an H-reflex facilitation, the timing of the major facilitation was shifted ∼15 ms earlier. This is expected, as the tap was generated within 1 ms, while the muscle response to stimulation took an average of 24.4 ms to become maximal. This finding is therefore consistent with the hypothesis that mechanical activation of ECR Ib afferents mediates the H-reflex facilitation.

Group Ib pathways in the upper limb are thought to be facilitated by cutaneous stimuli (Cavallari et al., 1985); we accordingly tested whether cutaneous stimulation to the dorsal side of digits 2 and 3 could modify the H-reflex facilitation. When the cutaneous stimulus was given 25 ms before the median nerve stimulus, the H-reflex amplitude was unchanged (Fig. 3*F*, H + cut; *p* = 0.2510). As previously shown, ECR conditioning stimulation 30 ms before the median nerve stimulus facilitated the H-reflex (Fig. 3*F*, H + ECR; *p* = 0.0101). ECR stimulation combined with cutaneous stimulation also caused facilitation of the H-reflex (Fig. 3*F*, H + ECR + cut; *p* = 0.0114); this facilitation was significantly larger than that following ECR stimulation alone (*p* = 0.0424). The enhancement of the facilitation by cutaneous stimuli is further evidence consistent with mediation by Ib pathways.

**Figure 4.**
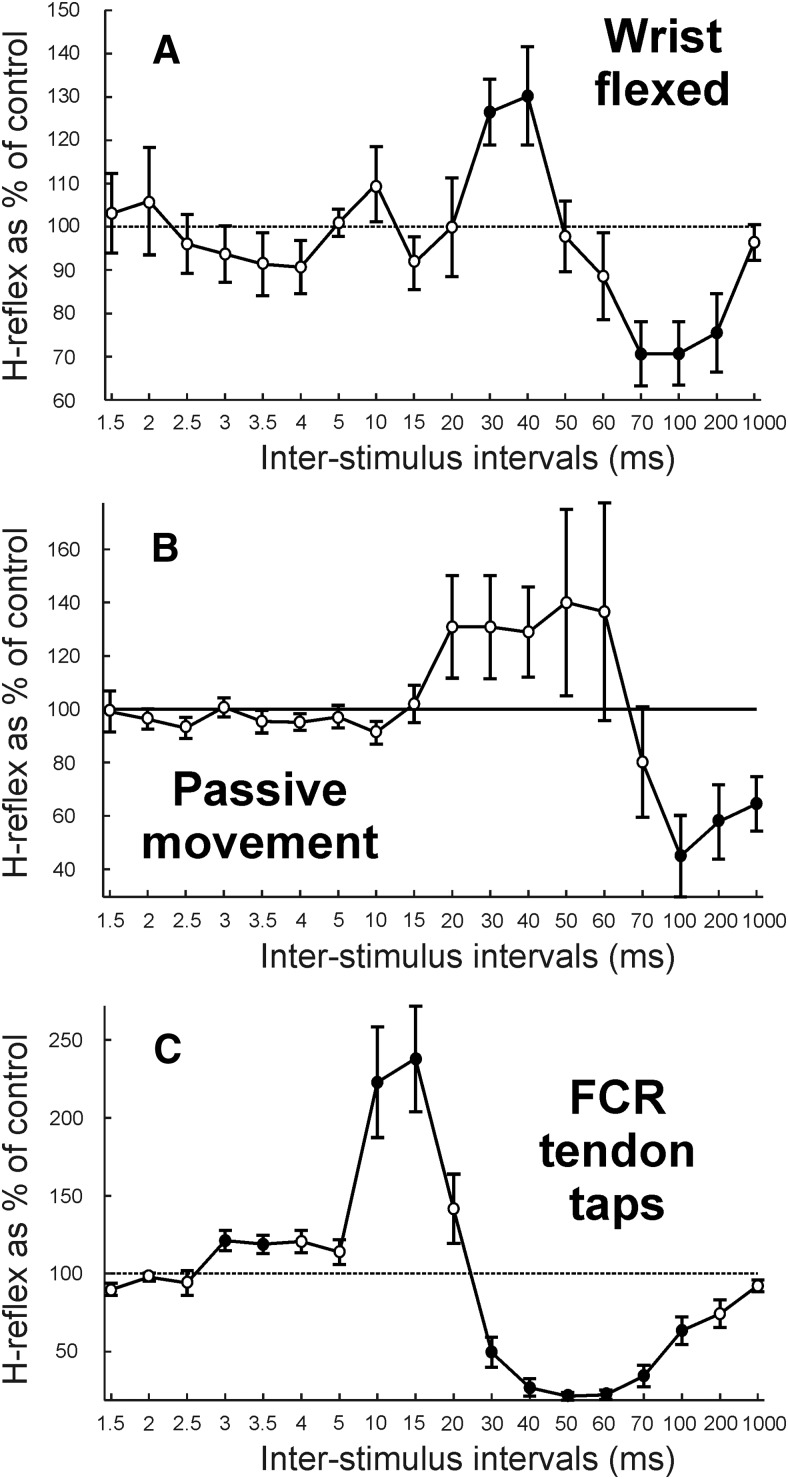
Studies in healthy humans probing which afferents mediate the facilitation. ***A***, Average from eight subjects of H-reflex amplitude with 18 ISIs between median nerve and conditioning ECR electrical stimulation at 3× MT, as in Figure 2, but while subjects had their wrist held in a flexed position. For one subject, data from seven ISIs (up to 5 ms) are not displayed as part of the average results due to stimulus artifact contamination. ***B***, Average from six subjects of H-reflex amplitude with 18 ISIs between median nerve and conditioning passive wrist extension movement caused by mechanical perturbation. ***C***, Average from five subjects of H-reflex amplitude with mechanical tap to FCR tendon as conditioning stimulation at 1× TT. Display conventions are as in Figure 2.

An alternative hypothesis for the generation of the facilitation is that the extensor muscle twitch generates a wrist extension, which thereby stretches the wrist flexor muscles and activates group Ia and/or Ib afferents originating from flexors. We first investigated this by repeating the main protocol while subjects had their wrist held in a flexed position, which disengaged the FCR muscle and should have greatly reduced flexor afferent activation after the ECR twitch. There was a significant effect of ISI (ANOVA, *F*_(17,112)_ = 3.65, *p* < 0.001). Figure 4*A* shows that there was still a significant facilitation at ISIs of 30 and 40 ms (*t* test, *p* = 0.0138 and *p* = 0.0421, respectively) and significant suppression at ISIs of 70, 100, and 200 ms (*t* test, all *p* < 0.040). The facilitation at the 30 ms ISI was smaller than with the wrist in a neutral position, but the comparison between the two using a two-sample *t* test did not reach significance (*p* > 0.05). This suggests that FCR afferents cannot be the only source of the facilitation effect.

To clarify further whether afferents activated by FCR stretch might play an important role in the facilitation, we repeated the main protocol replacing the ECR electrical stimulation by a passive extension movement of the wrist. This movement was substantially larger than the movement produced after the ECR shock at 3× MT. We expect that the passive movement would activate flexor afferents strongly due to the muscle stretch, but cause little or no activation of extensor Ib afferents. Figure 4*B* shows average results across subjects. There was a significant effect of ISI (ANOVA, *F*_(17,85)_ = 2.53, *p* = 0.002). A small but nonsignificant facilitation was seen for ISIs of 20–60 ms, and a significant suppression for ISIs of 100–1000 ms (*t* test, all *p* < 0.040). Again, this result suggests that stretch activation of FCR afferents is unlikely to be the only source of the facilitation at the 30 ms ISI.

Finally, we tested the effect of replacing the shock to ECR by a mechanical tap to the FCR muscle tendon, which should generate predominant activation of FCR afferents. There was a significant effect of ISI (ANOVA, *F*_(17,68)_ = 16.95, *p* < 0.001). Figure 4*C* shows average results indicating significant facilitation at the ISIs of 3, 3.5, 10, and 15 ms (*t* test, all *p* < 0.050) and significant suppression for ISIs of 30–100 ms (*t* test, all *p* < 0.030). This implies that the facilitation can be produced by the activation of FCR afferents alone, without the involvement of ECR afferents.

### Monkey experiments

**Figure 5.**
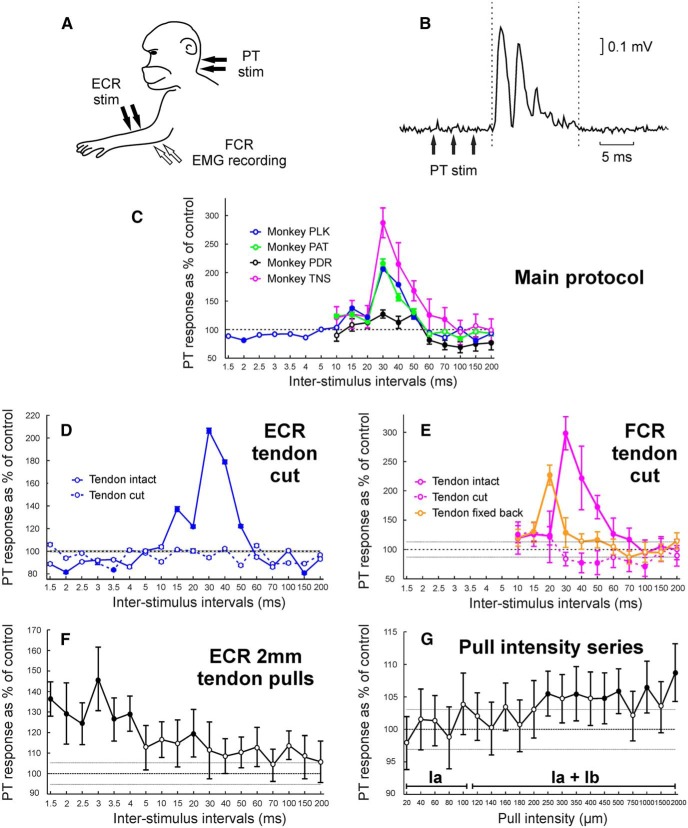
Monkey experiments. ***A***, Schematic representation of experimental setup. Wire electrodes for EMG recording were inserted into the FCR muscle, and for stimulation (stim) into the ECR muscle. Contralateral PT electrodes were used to evoke responses in the FCR. ***B***, Example of FCR response (rectified EMG signal) to PT stimulation from monkey PDR. Trains of three stimuli, 3 ms apart, were used to evoke responses in this monkey. Dashed lines indicate the region used to calculate the area under the curve as a measurement of PT response amplitude. ***C***, Main protocol results from monkeys PLK (blue), PAT (green), PDR (black), and TNS (pink). For each monkey, the average PT response amplitude is shown for 18 ISIs using conditioning ECR electrical stimulation at 3× MT. For monkeys PAT, PDR, and TNS data from ISIs up to 5 ms are not displayed due to stimulus artifact contamination. ***D***, Results are as in ***C***, with ECR tendon intact (continuous blue line) and cut (dashed blue line). Monkey PLK. ***E***, Results as in ***C***, with FCR tendon intact (continuous pink line), cut (dashed pink line), and reattached (continuous orange line). Monkey TNS. For ***D–G*** error horizontal lines, either side of 100% (control H-reflex) represent the SE of all repetitions of the control H-reflex (with no conditioning stimulation); for ***D*** and ***E***, these error lines represent the average control H-reflex error across all datasets displayed. ***F***, Average PT response amplitude as the percentage of control for 18 ISIs, using 2-mm-amplitude mechanical pulls to the ECR tendon as the conditioning stimulus. Monkey PDR. Note that for this protocol the responses are synchronized to the first of three shocks given to PT instead of the last shock, as used in other protocols. ***G***, Average PT response amplitude as the percentage of control, as a function of ECR tendon pull amplitude used as the conditioning stimulus (ISI, 2.5 ms). Bar above the abscissa indicates intensities expected to activate different afferent classes based on the study by Lundberg and Winsbury (1960). Display conventions for ***C–G***, as in Figure 2.

We tested the main protocol in all four monkeys, conditioning the response to PT stimulation with prior stimulation of the ECR muscle (Fig. 5*A*,*B*). Figure 5*C* shows results excluding data contaminated by stimulus artifact (ISIs up to 5 ms for monkeys PAT, PDR, and TNS). There was a significant effect of ISI for all four monkeys (ANOVAs: monkey PDR: *F*_(10,209)_ = 38.47, *p* < 0.001; monkey PLK: *F*_(17,342)_ = 13.9, *p* < 0.001; monkey PAT: *F*_(10,209)_ = 10.46, *p* < 0.001; monkey TNS: *F*_(10,209)_ = 37.36, *p* < 0.001). Although the strength and temporal extent of the effects varied between monkeys, in all cases there was a facilitation at the 30 ms ISI similar to that observed in the human studies.

We repeated the main protocol in three monkeys after cutting the ECR tendon near the wrist. In this preparation, electrical activation of afferents by the stimulus should persist, but the stimulus-evoked twitch should be prevented from activating ECR Ib afferents, or generating a wrist movement that could stimulate flexor afferents. Figure 5*D* shows the results in monkey PLK; the tendon cut had a significant effect on the curve (ANOVA, *F*_(1,684)_ = 25.3, *p* < 0.001). Following the tendon cut, the facilitation for ISIs ∼30 ms was lost. Results from the other monkeys in this protocol were similar. For monkey PAT, a significant effect of tendon cut on the curve was observed (ANOVA, *F*_(1,418)_ = 21.1, *p* < 0.001); after cutting the ECR tendon, the facilitation at the 30 ms ISI was lost, and there was significant facilitation only at the 10, 15, and 20 ms ISIs, which was also present with tendon intact (all *t* tests, *p* < 0.050), and significant inhibition at the 70 ms ISI (*p* = 0.0497), which was not present with tendon intact (data not shown). For monkey PDR, although a visible peak at the 30 ms ISI was lost after cutting the ECR tendon, ANOVA on the effect of tendon cut did not reach significance, *F*_(1,418)_ = 1.99, *p* = 0.158).

To test the hypothesis that FCR afferents were involved in the facilitation effect, we repeated the main protocol in monkey TNS after cutting the FCR tendon near the wrist and subsequently after reattaching it. If ECR Ib afferents alone were causing the facilitation with no involvement of FCR afferents, it would be expected that cutting the FCR tendon would not change the effect. Figure 5*E* shows that the broad facilitation of the ∼30 ms ISI was lost, or even reversed to a suppression, following the FCR tendon cut. When the tendon was reattached to the hand so that wrist extension once again generated muscle stretch in FCR, the facilitation was restored, although it was of lower amplitude and peaked earlier (at the 20 ms ISI) than originally. The effects were significant (ANOVA, factor tendon intact/cut/reattached, *F*_(2,627)_ = 79.98, *p* < 0.001). These results imply that FCR afferents also play an important role in the generation of the facilitation of the ∼30 ms ISI.

To investigate whether we could see the facilitation after activating ECR afferents without involvement of flexor afferents, we reproduced the main protocol in one monkey, replacing the conditioning stimuli by strong mechanical pulls on the cut ECR tendon. There was a significant effect of ISI (ANOVA, *F*_(17,342)_ = 2.2, *p* < 0.004). Figure 5*F* shows that a significant facilitation was generated at 1.5–4 ms ISIs (*t* test, *p* < 0.05). Note that for this protocol, the intervals quoted are times until the first stimulus of the train of three to the PT, instead of the last stimulus, as was used for other protocols; these intervals therefore correspond to 7.5–10 ms before the last (effective) PT stimulus. This indicates that a facilitation could be produced solely by ECR afferent activation.

Subsequently, to investigate which category of ECR afferents might generate the facilitation, we tested a series of tendon pulls of different amplitudes in the same monkey for a fixed ISI of 2.5 ms; the result is illustrated in Figure 5*G*. There was a significant effect of pull intensity (ANOVA, *F*_(19,1980)_ = 1.88, *p* = 0.011). Significant facilitations were seen only for pulls >250 μm (*t* test, *p* < 0.05). Lundberg and Winsbury (1960) reported that muscle stretches up to 100 μm activate Ia afferents exclusively, while stretches of >100 μm also activate Ib afferents. Our results suggest that the facilitation was not generated at intensities consistent with the activation of Ia afferents alone; only amplitudes expected to activate Ib afferents produced facilitation.

## Discussion

**Figure 6.**
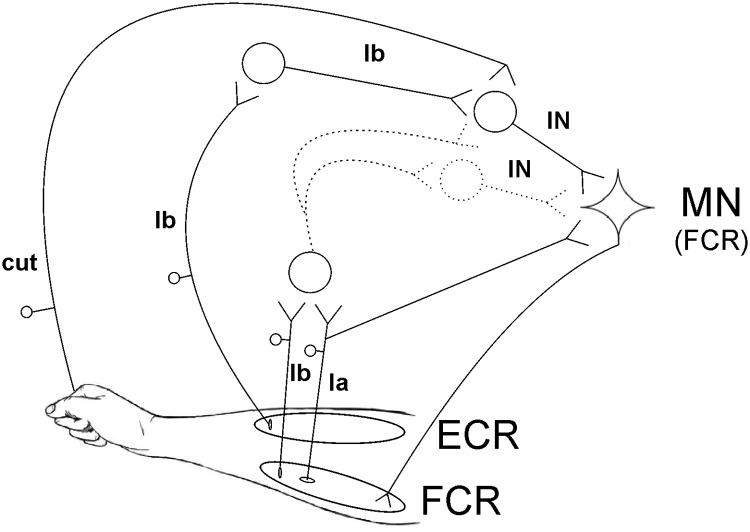
Schematic representation of the spinal circuits causing FCR H-reflex facilitation. Two convergent inputs excite the motoneurons (MN) of the FCR muscle, one originating from ECR Ib afferents and the other from Ib and/or Ia afferents in FCR. ECR Ib afferents synapse with Ib interneurons and subsequently with another set of interneurons (IN; pathway described by Pierrot-Deseilligny and Burke, 2012). It is uncertain whether input from FCR afferents converge onto the same interneurons as the input from the ECR Ib afferent or onto another set of interneurons. Cutaneous (cut) inputs facilitate Ib pathways.

In this study, we conditioned the FCR H-reflex with electrical stimulation over the ECR muscle belly and reported a reflex facilitation at 30 ms ISI. Our findings reveal novel spinal circuits, easily assessed noninvasively in humans. Some properties of these circuits are schematically illustrated in Figure 6, which may form a useful reference as individual features are described.

### Afferent pathways mediating facilitation

Several lines of evidence indicated that the facilitation was generated by the mechanical consequence of the ECR stimulus. The lack of changes in threshold intensity after prolonged vibration (Fig. 3*B*) argued against a contribution from electrical activation of ECR Ia afferents. Electrical activation of Ib afferents at the Golgi tendon organ was unlikely, given their location at the musculotendinous junction far from the stimulus site, although it remains possible that these afferents could have been activated at the point where the nerve leaves the muscle. The twitch time of the muscle (Fig. 3*C*,*D*) was consistent with the timing of the facilitation effect, which peaked at the 30 ms ISI. The facilitation came earlier when the sluggish ECR twitch was replaced with a rapid mechanical tap or pull to the muscle (Figs. 3*E*, 5*F*). Finally, the facilitation was lost when the ECR tendon was cut (Fig. 5*D*), which would prevent the muscle from generating mechanical effects but leave electrical activation of afferents within the ECR unchanged.

It is well known that electrical stimulation of a muscle powerfully activates group Ib afferents (Hunt and Kuffler, 1951). Multiple findings indicated a contribution to the facilitation from ECR Ib afferents. The facilitation was preserved when the wrist was flexed (Fig. 4*A*), which would tend to disengage the wrist flexor muscles and prevent activation of flexor afferents. Although the facilitation could be generated by taps or pulls to ECR (Figs. 3*E*, 5*F*), the threshold pull intensity was above that reported to activate only group Ia afferents (Fig. 5*G*), suggesting a requirement for the activation of Ib afferents, which are known to be discharged by tendon taps in humans (Burke et al., 1983). Finally, the facilitation could be augmented by suitably timed cutaneous stimulation (Fig. 3*F*). Cavallari et al. (1985) reported that a cutaneous shock placed 7 ms before the median nerve stimulus could facilitate putative Ib pathways; no other intervals were tested. Our study used a longer interval of 25 ms, which was chosen based on a pilot study to give the most robust effect. The electrically evoked twitch would activate ECR Ib afferents with a much more dispersed time course compared with the temporally precise radial nerve stimulus used by Cavallari et al. (1985). It is therefore most likely that we accessed the same cutaneous pathway.

The second possibility that we investigated was that the ECR twitch produced a wrist extension movement, which stretched the wrist flexor muscles and activated flexor afferents. This mechanism must play some role. Tendon taps to the FCR muscle could also generate H-reflex facilitation (Fig. 4*C*). Cutting the FCR tendon abolished the facilitation, which was restored if a connection between tendon and wrist movement was restored (Fig. 5*E*). Passive wrist extension by a torque motor also seemed to generate a weak facilitation (Fig. 4*B*), although this was heterogeneous across subjects and failed to reach significance. Group Ia afferents in flexors would be the most obvious candidates to mediate this effect, but a contribution from Ib afferents is also possible.

Other alternatives for the afferents mediating the facilitation seem improbable. The surface stimulus over ECR in human subjects would undoubtedly activate cutaneous receptors, but the facilitation was also seen in the monkey experiments using intramuscular wires, so a cutaneous origin is unlikely. Group Ia afferents in ECR are also unlikely to contribute. These afferents pause their firing after a twitch evoked by electrical stimulation, as the muscle goes slack (Hunt and Kuffler, 1951). Pulling the ECR tendon with amplitudes of <100 μm, which should activate Ia afferents exclusively, did not cause facilitation. As noted above, direct electrical activation of Ia afferents in ECR was unlikely to be the origin of the effect as the effect was lost after cutting the ECR tendon, and the threshold for the facilitation was unaltered by prolonged vibration, which should raise the electrical threshold for the stimulation of group Ia fibers.

Our evidence thus suggests that both ECR Ib afferents and FCR afferents participate concurrently in producing the H-reflex facilitation in FCR. It remains uncertain whether these afferents connect to common or separate interneurons. We cannot determine definitively what central pathway generates this effect, as the dispersed time course of afferent activation after ECR stimulation precludes precise measures of central delay. One possibility is that it involves nonsegmental circuits, such as C3–C4 propriospinal interneurons. However, the peak of the facilitation occurs rapidly after the peak of the twitch tension in the ECR (Fig. 3*D*), suggesting a short central delay, which is consistent with mediation by segmental interneurons.

Work in cats has suggested that the excitatory Ib pathway is trisynaptic: the afferents excite Ib interneurons, which subsequently synapse with a further set of interneurons, which in turn excite motoneurons (Pierrot-Deseilligny and Burke, 2005). In our anesthetized monkey recordings, we found that the amplitude of facilitation produced by the same stimulus could vary between separate recordings (Fig. 5, compare *F*, *G* for points in the 2.5 ms interval and the 2000 μm amplitude). This is in agreement with the existence of a trisynaptic pathway, which would be expected to be especially sensitive to the fluctuations in anesthetic depth that typically occur in such studies. Our results suggest that for the primate wrist, this putative circuit receives highly converging inputs (Fig. 6).

As well as demonstrating inputs from afferents in ECR and FCR muscles, plus cutaneous receptors in the digits, we also showed in some pilot experiments (not reported here) that a similar FCR H-reflex facilitation could follow taps to the tendon of extensor carpi ulnaris and flexor carpi ulnaris. In cat lower limb, the effect of Golgi tendon organs on antagonist muscles can switch from inhibition to excitation during locomotion (Pearson and Collins, 1993; Prochazka et al., 1997), providing flexible sensory feedback. How Golgi tendon organs act during voluntary movements of the human upper limb is yet to be determined, but we assume that the pathway we describe plays an important role. With wrist flexed, the H-reflex facilitation should be mediated primarily by ECR afferent Ib rather than FCR afferents. This posture might therefore be used to maximize the specificity of our protocol for noninvasive assessment in humans.

The monkey and human experiments differed in several important regards, even though motor circuits in these species are very similar. The monkeys were anesthetized, whereas the human volunteers were awake and at rest. Anesthesia will suppress central circuits, although our experience with microelectrode recordings under this anesthetic combination is that spinal interneurons remain highly active. Different test stimuli were used in the two species, but both will generate monosynaptic inputs to FCR motoneurons, giving an assessment of motoneuron excitability. Nonmonosynaptic input pathways may also contribute, although only minimally, for PT stimulation, where such circuits are dominated by feedforward inhibition (Maier et al., 1998; Alstermark et al., 1999). Synaptic input from Ia afferents is subject to presynaptic inhibition (Rudomin and Schmidt, 1999), but corticospinal input is not (Nielsen and Petersen, 1994; Jackson et al., 2006). Despite these differences, remarkably similar facilitations following ECR stimuli could be seen in data from humans and monkeys (compare Figs. 1*C*, 2 with Fig. 5*C*). This suggests that both facilitations largely reflect postsynaptic increases in motoneuron excitability, rather than presynaptic or upstream effects.

### Comparison with effects of conditioning stimuli to the radial nerve

When the FCR H-reflex is conditioned by stimulation of the radial nerve at the spiral groove, three phases of reflex suppression are typically seen (Day et al., 1984). The short-latency effect (intervals −1 to +3 ms) is suggested to arise from disynaptic inhibition caused by either group Ia or Ib extensor afferents (Day et al., 1984; Wargon et al., 2006). We also found evidence for a suppression at short intervals by ECR stimulation (3–5 ms; Fig. 2), which could be mediated by the same pathway—the slightly longer latency is compatible with the more distal location of the ECR stimulation site compared with the radial nerve in the spiral groove. The suppression was smaller than previously reported using radial nerve stimulation—a <20% reduction (Fig. 2)—and was not consistently observed in all subjects (Fig. 1*C*). Stimulation of the radial nerve at short intervals reduces the H-reflex by ∼75% (Day et al., 1984). This may be because fewer afferents were stimulated within the single muscle compared with the whole nerve. Additionally, intramuscular stimulation may be more likely to activate group Ia than Ib afferents directly, given the location of muscle spindles within the muscle belly compared with Golgi tendon organs at the musculo–tendon junction (Matthews, 1972). It has been suggested that short-latency disynaptic inhibition at the wrist is predominantly dependent on Ib afferents (Wargon et al., 2006), which could also explain why smaller effects at short latency were seen in our studies.

Two later phases of suppression following radial nerve stimulation, at 5–50 and 50–1000 ms, are referred to as D1 and D2 inhibition (Mizuno et al., 1971). D1 inhibition most likely arises from presynaptic inhibition of the FCR group Ia afferents (Berardelli et al., 1987); the origin of D2 inhibition is less certain, although it may also be presynaptic (Lamy et al., 2010). We found a clear long-lasting suppression that peaked ∼70 ms ISI; this could be comparable to the previous reports of D2 inhibition, although it is beyond the scope of the current report to investigate this further.

One intriguing question is why we observe reflex facilitation at ISIs of ∼30 ms when conditioning using ECR stimulation, whereas stimulating the radial nerve generates a D1 suppression at these intervals. It is possible that this reflects a difference in the relative proportions of different afferents activated. Although intramuscular stimulation can activate group I afferents directly (Muir and Lemon, 1983), our results after prolonged vibration suggest that electrical activation of Ia afferents contributes little to the facilitation at 30 ms. The ECR muscle twitch likely produces a very powerful Ib afferent activation (Hunt and Kuffler, 1951). The different effects at 30 ms ISIs between radial nerve and ECR stimulation may therefore reflect a dominance of activation of group Ia afferents (producing D1 suppression) or group Ib afferents (producing facilitation), respectively. However, in conflict with this argument is the observation that a tap delivered to the ECR tendon also produced a facilitation (Fig. 3*E*); we would expect a tendon tap to activate group Ia afferents more powerfully than group Ib afferents (Burke et al., 1983). One important factor may be that both ECR tendon taps and ECR twitch are likely to produce repetitive afferent discharge (Hunt and Kuffler, 1951; Burke et al., 1983), which may alter the relative balance between suppression and facilitation. It is known, for example, that presynaptic inhibition is weaker if measured using a tendon tap reflex than an electrically evoked H-reflex, presumably reflecting a difference between effects on repetitive versus a single spike discharge (Morita et al., 1998).

In summary, we have described a spinal circuit with converging input from wrist flexor afferents and extensor Ib afferents onto the wrist flexors (Fig. 6). This circuit can be easily assessed noninvasively in humans with the measurement of the FCR H-reflex conditioned by ECR stimulation. Such a finding represents a contribution to our knowledge of human spinal cord circuitry as well as having potential clinical relevance in aiding the understanding of changes occurring after damage, such as following stroke and spinal cord injury.
